# Highly efficient biomethane production from chicken manure and municipal organic solid waste using magnetite: converting waste into energy

**DOI:** 10.1007/s10532-026-10249-2

**Published:** 2026-02-07

**Authors:** Tuğçe Bay, Buğse Büşra Vural, Öznur Begüm Gökçek

**Affiliations:** 1 Department of Environmental Engineering, Faculty of Engineering, Niğde Ömer Halisdemir University, 51100 Niğde, Türkiye; 2 Department of Energy Science and Technologies, Niğde Ömer Halisdemir University, Niğde, Türkiye

**Keywords:** Chicken manure, Biogas yield, Magnetite, Biomethane production

## Abstract

The aim of this study is to investigate the effect of magnetite (Fe_3_O_4_) addition on biogas and biomethane production in the anaerobic treatment of chicken manure (CM) and municipal organic solid waste (MOSW). Batch experiments were conducted under mesophilic conditions using different substrate-to-inoculum (S/I) ratios (0, 1, 2, and 4 g VS-S/g VS-I) and magnetite concentrations (50, 100, 200, 400, and 600 mg L⁻^1^). The highest biogas and biomethane production was obtained in the S/I = 1 gVS-S/gVS-I, 2:1 (CM: MOSW) reactor and were 2910.5 ± 199.4 mL CH_4_/gVS and 1718.03 ± 117.73 mL CH_4_/gVS, respectively. At different magnetite concentrations, the highest biogas and biomethane production occurred at 200 mgL^−1^ magnetite loading rate, 1842.7 ± 112.0 mL CH_4_/gVS and 1081.99 ± 65.78 mL CH_4_/gVS, respectively. The highest total organic carbon (TOC) and total nitrogen (TN) concentrations were determined at S/I = 4, 2:1 (CM: MOSW) gVS-S/gVS-I loading ratio, while the highest TS and VS removal efficiency was determined at S/I = 1 gVS-S/gVS-I, 2:1 (CM: MOSW) ratio and 100 mgL^−1^ magnetite loading ratio. When the microbial distribution was examined, the first five dominant species (*W5, S1, Coprothermobacter, Treponema* and *Fervidobacterium*) did not change after the addition of magnetite. The findings demonstrate the positive effects of magnetite addition on biogas and biomethane production, providing significant insights for the development of new strategies to enhance anaerobic digestion processes.

## Introduction

The global community faces escalating energy demand, increasing greenhouse gas emissions, and intensifying climate change impacts, underscoring the urgent need for sustainable energy solutions. In this context, renewable energy systems based on biomass conversion have gained increasing attention due to their potential to reduce fossil fuel dependency and mitigate greenhouse gas emissions (Ma et al. 2021; Abanades et al. 2022).

Anaerobic digestion is a well-established biochemical process that converts organic matter into biogas and methane through a series of microbial reactions under oxygen-free conditions. Due to its effectiveness in treating organic-rich wastes and wastewater, anaerobic digestion has been widely implemented for energy recovery and waste management worldwide (Lohani et al. 2021; Liu et al. 2021) The process involves sequential steps of hydrolysis, acidogenesis, acetogenesis, and methanogenesis and supports multiple energy applications, including electricity and heat generation, combined heat and power systems, and biomethane upgrading (Abanades et al. 2022).

A wide variety of feedstocks can be utilized for anaerobic digestion, including agricultural residues, animal manure, municipal solid waste, and food processing by-products. Chicken manure (CM), one of the substrates examined in this study, represents a significant organic waste stream, with individual chickens producing approximately 0.08–0.1 kg of manure per day (Manogaran et al. 2023). While its high nitrogen content poses challenges related to ammonia inhibition, CM remains a promising substrate for anaerobic digestion when appropriately managed (Yin et al. 2021). Municipal organic solid waste (MOSW), another substrate used in this study, is characterized by high biodegradability and moisture content, making it well suited for anaerobic treatment and contributing to reduced environmental and public health impacts (Gökçek et al. 2021).

One of the key advantages of anaerobic digestion is its flexibility, allowing the co-digestion of multiple substrates and the use of additives to enhance process performance (Adedeji et al. 2022). In recent years, conductive materials have attracted considerable interest as additives due to their ability to enhance microbial interactions and improve methane production. Iron-based conductive materials such as magnetite have been shown to support syntrophic cooperation between fermentative bacteria and methanogenic archaea, thereby improving digestion efficiency (Baniamerian et al. 2019; Ajay et al. 2020; Nabi et al. 2022).

The enhancement effect of magnetite is often attributed to its role in facilitating direct interspecies electron transfer (DIET), which promotes more efficient electron exchange between microbial partners and improves methane production rates. Previous studies have reported increased methane yields and organic matter removal with magnetite supplementation across various substrates, including municipal waste, wastewater, and agricultural residues (Wang et al. 2018; Zhang et al. 2018; Lei et al. 2018; Khalid et al. 2019). Similar performance improvements have also been observed with other conductive materials such as biochar, graphene oxide, and activated carbon (Muratçobanoğlu et al. 2022; Al Hasani et al. 2022; Ngo et al. 2023).

Despite these advances, existing studies often investigate either conductive material addition or substrate loading conditions in isolation, without systematically examining their combined effects or linking process performance to microbial community behavior. In particular, limited attention has been given to identifying optimal interaction windows in which substrate-to-inoculum ratios and conductive material dosage jointly influence methane production and process stability.

In this context, the present study investigates the combined effects of substrate loading conditions and magnetite supplementation on biogas and biomethane production from chicken manure and municipal organic solid waste. By integrating process performance indicators with microbial community analysis, this work demonstrates that magnetite effectiveness is strongly dependent on substrate-to-inoculum ratio and dosage control. The results reveal a distinct operating window in which methane yield and volatile solids removal are maximized without inducing major shifts in microbial community structure. These findings provide new insights into the functional role of conductive additives in anaerobic digestion systems and offer practical guidance for the stable and efficient treatment of nitrogen-rich wastes, supporting circular economy strategies and sustainable energy transition.

## Material and methods

### Inoculum and substrate

Fresh chicken manure was collected from the Niğde Ömer Halisdemir University Chicken Farm, while municipal organic solid waste (MOSW) was obtained from the regular storage facility managed by the Niğde Municipality Solid Waste Union. The anaerobic inoculum sludge was sourced from the anaerobic digester unit of the Kayseri Advanced Biological Wastewater Treatment Plant, which operates under mesophilic conditions. The organic fraction of the domestic waste supplied by the Niğde Municipality was manually separated and subsequently stored at − 18 ℃ to preserve its composition and integrity. Before utilization, both chicken manure and MOSW were shredded to a maximum particle diameter of 1 cm to achieve homogeneity, enhance surface area, and facilitate biodegradation. he inoculum sludge was gently homogenized by stirring before use to ensure uniformity. It was then pre-incubated at 37 ℃ for 5 days to reduce residual biogas production and stabilize microbial activity. During storage, the inoculum was maintained at 4 ℃ to minimize shifts in the microbial community. To characterize the substrates, analyses of total solids (TS), volatile solids (VS), total organic carbon (TOC), total nitrogen (TN), carbon-to-nitrogen (C/N) ratio, and pH were conducted according to standard analytical methods. The physicochemical characteristics of chicken manure, municipal organic solid waste, and anaerobic inoculum sludge are summarized in Table 1.

**Table 1:** Physicochemical properties of chicken manure, municipal solid waste, and anaerobic inoculum sludge

	Chicken manure	Municipality solid waste	Anaerobic inoculum sludge
TS (%)	25.75	30	30.53
VS (%)	18.35	28	19.06

### Experimental design

In this study, inoculum sludge, chicken manure, and MOSW were introduced into 500 mL batch reactors at varying substrate-to-inoculum (S/I) ratios expressed in gVS-S/gVS-I. The ratios 1:1, 2:1, and 1:2 indicate the respective proportions of chicken manure (CM) and municipal organic solid waste (MOSW) used in the reactors. To evaluate the impact of magnetite supplementation on biogas and biomethane production, different dosages were applied at the optimum S/I ratio determined in the experimental process. Each batch reactor was loaded with 300 mL of anaerobic inoculum sludge, followed by the homogeneous addition of CM and MOSW based on the pre-calculated gVS amounts. To ensure anaerobic conditions, the reactors were flushed with N₂ gas for 1 min to remove residual oxygen, sealed with airtight silicone lids, and subsequently incubated at 37 ℃ oven for temperature regulation. The data are expressed as mean values with standard deviation. Magnetite (iron(II,III) oxide, Fe₃O₄; 97% metals basis) used in this study was purchased from Alfa Aesar (product no. 12374). The material was used as received without any chemical pretreatment. Prior to reactor addition, magnetite was homogenized by gentle mixing to minimize particle aggregation and directly added to the reactors at the specified concentrations. No adverse effects related to magnetite settling or instability were observed during the digestion period, indicating stable behavior of the material under anaerobic conditions. All anaerobic digestion experiments were conducted under controlled batch conditions using replicate reactors (n = 3), and the reported results represent mean values of the replicates. Experimental deviations among replicates were within acceptable ranges and did not affect the observed trends.

### Analytical methods

Following reactor setup, methane (CH₄) analysis was initiated using a GC Agilent Technologies 7820A gas chromatograph. The system was equipped with an Agilent CRB XEN column. The oven program started at 100 ℃ for 1 min, followed by a temperature ramp of 20 ℃ min⁻^1^ to 220 ℃, where it was maintained for 6 min. The injector and detector temperatures were set to 115 ℃ and 230 ℃, respectively. Helium was used as the carrier gas at a constant flow rate of 30 mL min⁻^1^, and 200 µL biogas samples were injected at predefined intervals throughout the experiment. Methane concentrations were measured daily during the first five days, every three days during the subsequent fifteen days, and every five days thereafter. Biogas production was quantified using the displacement method with a glass syringe. Upon opening the reactors, calibrated probe tips were immersed directly into the homogenized samples for measurement. pH and oxidation–reduction potential (ORP) analyses were performed using a WTW Multi 3620 IDS device. Total solids (TS) and volatile solids (VS) analyses were conducted according to SM 2540 D and SM 2540 standard methods (APHA 2005), while total organic carbon (TOC) and total nitrogen (TN) analyses were performed using a Shimadzu TOC analyzer equipped with an SSM module. All TOC, TN, TS, and VS analyses were performed at the end of the digestion experiments to evaluate final substrate degradation and removal efficiencies.

### Microbial community analysis

In the experiment conducted to define the optimal substrate-to-inoculum (S/I) ratio, the highest methane yield was recorded at an S/I = 1, 2:1 gVS-S/gVS-I (CM:MOSW = 2:1) reactor, as well as in the reactor supplemented with 200 mg L⁻^1^ magnetite. To examine the microbial community structure, approximately 10 mL of fermenter sludge (bulk sludge biomass) was collected at the end of the experiments (day 63 for S/I = 1 and 2:1 gVS-S/gVS-I, and day 50 for the magnetite-supplemented reactors). The samples were stored at –20 ℃ until DNA extraction. DNA isolation was performed using the DNeasy PowerSoil Kit (QIAGEN) according to the manufacturer’s instructions, using 0.5 mL of sludge per extraction. The quantity and purity of the isolated DNA were assessed using a Quawell NanoDrop spectrophotometer (Q5000, Quawell UV–VIS Spectrophotometer, USA), ensuring that A260/280 ratios were within the range of 1.8–2.0. Variations in microbial community composition were analyzed using next-generation sequencing (NGS), specifically targeting the V3–V4 regions of the 16S rRNA gene. Sequencing was performed on the Illumina iSeq 100 platform (paired-end, 2 × 150 bp) using the iSeq 100 i1 Reagent Kit. The 16S rRNA gene sequencing and subsequent bioinformatic analyses were performed by an external service provider (RefGen Biotechnology). Raw reads were subjected to quality control using FastQC and trimmed with Trimmomatic to remove low-quality bases and adapters. The microbial community analysis presented in this study is based on processed taxonomic and diversity outputs provided by the service provider, as raw sequencing data were not available for public deposition. Taxonomic assignment was performed using the RDP Classifier against the Greengenes database, and operational taxonomic units (OTUs) were assigned accordingly. Sequencing yielded 155,110 and 178,561 high-quality reads, corresponding to an average sequencing depth of approximately 166,836 reads per sample after quality filtering, which was sufficient to reliably capture dominant microbial taxa. Alpha diversity was evaluated using the Shannon index (Hill et al. 2003) and Simpson index (Simpson 1949; Magurran 2004), and diversity calculations were performed in R using normalized genus-level relative abundance tables.

## Results and discussion

### Evaluation of biogas and biomethane production under different S/I ratios and magnetite concentrations

**Fig. 1:**
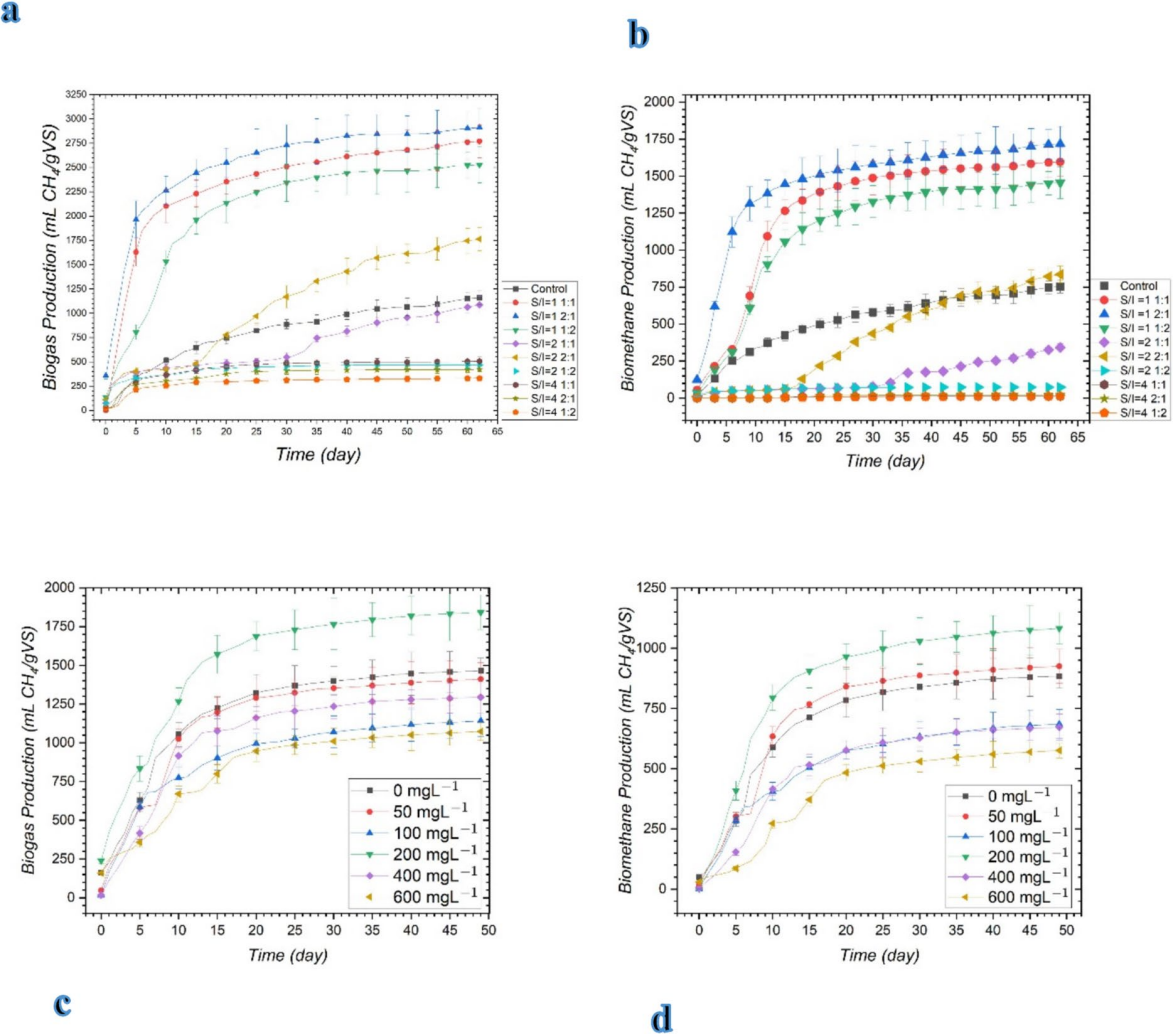
**a** Cumulative biogas yield at different S/I (gVS-S/gVS-I) ratios, **b** cumulative biomethane yield at varying S/I (gVS-S/gVS-I) ratios, **c** total biogas production at different magnetite concentrations, and **d** total biomethane production at different magnetite concentrations

Figure 1 presents the biogas and biomethane yields obtained from batch reactors operated under mesophilic conditions at different substrate-to-inoculum (S/I) ratios (g VS-S/g VS-I) and different magnetite concentrations. When biogas production at S/I ratios of 1, 2, and 4 g VS-S/g VS-I with CM:MOSW ratios of 1:1, 2:1, and 1:2 was compared, the highest biogas production was observed at S/I = 1 with a CM:MOSW ratio of 2:1 (2910.5 ± 199.4 mL CH₄/g VS), and the highest biomethane yield was recorded under the same operating condition (1718.03 ± 117.73 mL CH₄/g VS). As shown in Fig. 1, biogas and biomethane production yields were closely aligned, with the highest methane yield observed at the same loading condition that produced the maximum biogas yield. Accordingly, the optimum operating condition was identified as S/I = 1 g VS-S/g VS-I with a CM:MOSW ratio of 2:1. This finding is consistent with previous studies reporting that an S/I ratio of approximately 1 is favorable for maximizing methane yield in anaerobic co-digestion systems (Corsino et al. 2021; Owamah et al. 2021). At the optimum loading condition (S/I = 1, CM:MOSW = 2:1), reactors supplemented with different magnetite concentrations (50–600 mg L⁻^1^) showed the highest biogas production at 200 mg L⁻^1^ magnetite (1842.7 ± 112.0 mL CH₄/g VS), representing a 25.83% increase compared with the control reactor without magnetite addition (1464.3 ± 82.5 mL CH₄/g VS). Likewise, the highest methane yield was observed in the reactor supplemented with 200 mg L⁻^1^ magnetite (1081.99 ± 65.78 mL CH₄/g VS). This value represented a 22.39% increase compared to the control reactor without magnetite addition (883.93 ± 49.81 mL CH₄/g VS). These results clearly demonstrate that magnetite supplementation enhanced both biogas and methane production, with 200 mg L⁻^1^ identified as the most effective dosage. These findings suggest that magnetite at this dosage provided an optimal balance between enhanced microbial interactions and the avoidance of potential inhibitory effects observed at higher concentrations. In this system, the observed improvement can be attributed to the ability of magnetite to facilitate direct interspecies electron transfer (DIET) between fermentative bacteria and methanogenic archaea, thereby accelerating key metabolic conversion steps. This interpretation is supported by the observation that, although the dominant microbial taxa (*W5, S1, Coprothermobacter, Treponema, and Fervidobacterium*) remained largely unchanged following magnetite addition, methane yield increased by more than 22%. Thus, magnetite supplementation appears to have strengthened metabolic connectivity within the existing microbial community, rather than inducing major shifts in community composition. The observed improvements are consistent with previous studies reporting that conductive iron-based additives enhance methane yield by facilitating microbial electron transfer and stabilizing anaerobic digestion performance (Abdelsalam et al. 2017; Zhang et al. 2019; Aguilar-Moreno et al. 2020).

### Chemical and physical analysis parameters at different S/I loading ratios and magnetite concentrations

**Fig. 2:**
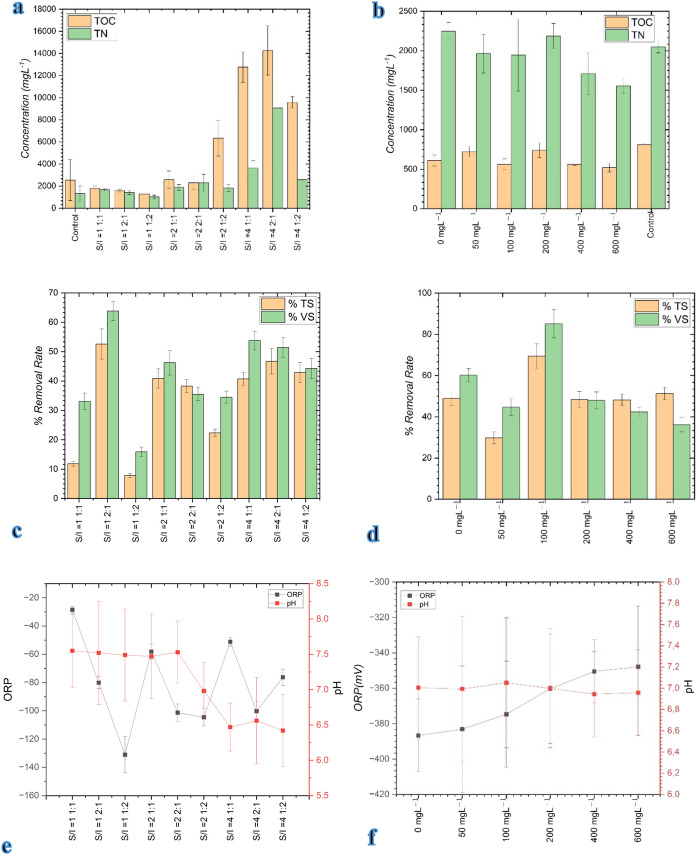
**a** TOC and TN measurements at various S/I (gVS-S/gVS-I) ratios, **b** TOC and TN measurements at different magnetite concentrations, **c** TS and VS removal efficiencies across various S/I (gVS-S/gVS-I) ratios, **d** TS and VS removal efficiencies at different magnetite concentrations, **e** pH and ORP levels at different S/I (gVS-S/gVS-I) ratios, and **f** pH and ORP levels at different magnetite concentrations

At different S/I (gVS-S/gVS-I) loading ratios, the highest TOC and TN concentrations were observed at S/I = 4, 2:1 gVS-S/gVS-I loading ratio and were 14 260.0 ± 2231.6mgL^−1^ TOC and 9068.0 ± 5908.6 mgL^−1^ TN, respectively. No significant difference was observed in TOC and TN values in reactors added with different magnetite concentrations compared to the control reactor without magnetite (Fig. 2a and b.). The elevated total nitrogen (TN) concentrations observed in the reactors are primarily attributed to the nitrogen-rich nature of chicken manure, which is characterized by high protein and uric acid content. Previous studies have reported that anaerobic digestion systems treating poultry manure under batch conditions commonly exhibit TN concentrations in the range of 6000–10,000 mg·L⁻^1^, particularly under high substrate loading rates (Yin et al. 2021; Manogaran et al. 2023). In the present study, the use of a CM-dominant substrate mixture (CM:MOSW = 2:1) combined with a substrate-to-inoculum ratio of 1 resulted in concentrated digestate conditions, leading to TN values approaching 9000 mg·L⁻^1^.

It is important to emphasize that TN represents the sum of organic and inorganic nitrogen species and does not directly indicate inhibitory ammonia levels. Ammonia inhibition in anaerobic digestion is primarily governed by free ammonia (NH₃), which is dependent on pH and temperature rather than total nitrogen alone (Chen et al. 2008; Rajagopal et al. 2013). Despite the elevated TN concentrations measured in this study, stable methane production profiles and the absence of performance deterioration indicate that ammonia inhibition did not occur under the applied operating conditions.

When the TS and VS experimental results were examined, the highest TS and VS removal efficiency was found at S/I = 1, 2:1 gVS-S/gVS-I ratio, which is the reactor with the highest biogas and biomethane production, and the TS and VS removal efficiencies were determined as 52.592 ± 5.130% and 63.841 ± 3.258%, respectively (Fig. 2c.). Similar to another study on this subject (Tariq et al. 2023), the highest TS and VS removal efficiency was obtained at the rate where the highest cumulative biogas yield was obtained. The amount of TS has a significant effect on the efficiency of anaerobic digestion, especially in terms of biogas and methane production, but also affects the microbial structure within the system (Kostopoulou et al. 2023). As a result of the addition of magnetite at different concentrations, the highest TS and VS removal efficiency was observed at 100 mgL^−1^ magnetite loading rate and was determined as 69.453 ± 6.015% and 85.047 ± 6.809%, respectively (Fig. 2d). These results indicate that magnetite supplementation promotes substrate degradation and enhances digestion efficiency, consistent with previous studies reporting improved TS and VS removal with magnetic additives (Tetteh et al. 2021).

The optimum pH range recommended for anaerobic treatment process is 6.8 to 7.2. However, AD process can tolerate 8.0 pH and affects biogas production since pH has a significant effect on microbial growth and production (Tariq et al. 2023). ORP (oxidation reduction potential) is an indicator of the capacity of molecules in wastewater or sludge to release or gain electrons. In this study, pH values were determined to be in the range of 6.42 ± 0.51- 7.55 ± 0.52 while ORP values ranged from − 28.5 ± 2.4 to − 131.1 ± 12.9 mV. It was determined that the pH dropped below 7 at the loading ratio of S/I = 4 gVS-S/gVS-I, and the pH was observed to be 7.5 ± at the loading ratios of S/I = 0, S/I = 1 and S/I = 2 gVS-S/gVS-I (Fig. 2e).

Across different magnetite concentrations, pH values were stable between 6.944 ± 0.401 and 7.051 ± 0.611, and ORP values ranged from − 347.9 ± 34.3 to − 386.7 ± 20.5 mV (Fig. 2f). At 200 mgL^−1^ magnetite—the condition with the highest methane yield—the pH was 7.000 ± 0.560 and ORP was − 360.3 ± 30.8 mV, both within the ranges previously reported for stable methanogenic activity (Mohammad Ali Abdoli 2016; Chotinath Vongvichiankul et al. 2017).

The dose-dependent response observed for magnetite supplementation indicates that its beneficial effect on anaerobic digestion operates within a limited operational window rather than following a linear trend. In the present study, a magnetite concentration of 200 mg L⁻^1^ resulted in the highest methane yield and volatile solids removal, whereas higher dosages led to reduced performance. Similar non-linear responses to magnetite and other conductive additives have been widely reported, where moderate concentrations enhance methane production while excessive dosages exert inhibitory effects (Abdelsalam et al. 2017; Baniamerian et al. 2019; Ajay et al. 2020).

At optimal concentrations, magnetite is known to provide conductive surfaces that facilitate electron transfer between syntrophic bacteria and methanogens, thereby enhancing methane formation efficiency (Kato et al. 2012; Baek et al. 2018). However, at elevated dosages, particle aggregation can occur, reducing the effective surface area available for microbial interaction and limiting conductivity benefits (Baniamerian et al. 2019). In addition, excessive iron oxide concentrations may alter local redox conditions or impose physical stress on microbial cells, potentially disrupting methanogenic activity (Park et al. 2018).

Although particle size distribution and surface chemistry were not directly measured in this study, the observed dose-dependent response is consistent with aggregation-related limitations reported for iron oxide additives under batch anaerobic digestion conditions.

Although direct electrochemical characterization was not performed in this study, indirect evidence supports this interpretation. Microbial community analysis revealed no significant shifts in dominant taxa following magnetite supplementation, despite substantial improvements in methane yield. Similar observations have been reported in previous studies, where conductive materials enhanced process performance without inducing major microbial community restructuring, suggesting improved metabolic efficiency within existing syntrophic networks rather than microbial selection (Cruz Viggi et al. 2014; Ajay et al. 2020).

Process indicators further support this explanation. Volatile fatty acid profiles did not show persistent accumulation under the optimal magnetite dosage, indicating that syntrophic conversion of intermediates was not impaired. Moreover, co-digestion of chicken manure and municipal organic solid waste improved the C/N balance of the system, mitigating ammonia-related stress—a known inhibitory factor in nitrogen-rich substrates (Abdelsalam et al. 2017; Baniamerian et al. 2019). Under these balanced conditions, the enhanced performance observed at 200 mg L⁻^1^ magnetite is therefore attributed primarily to improved process stability and electron transfer efficiency rather than to changes in substrate composition or microbial dominance. Therefore, the combined evaluation of process performance, stability indicators, and microbial structure supports a system-specific interpretation of magnetite-enhanced anaerobic digestion rather than a generalized DIET effect.

Overall, these findings demonstrate that magnetite acts as a conditional facilitator of anaerobic digestion, where dosage optimization is critical to avoid aggregation effects, redox imbalance, or inhibitory interactions that may negate its beneficial role.

### Microbial community

**Fig. 3:**
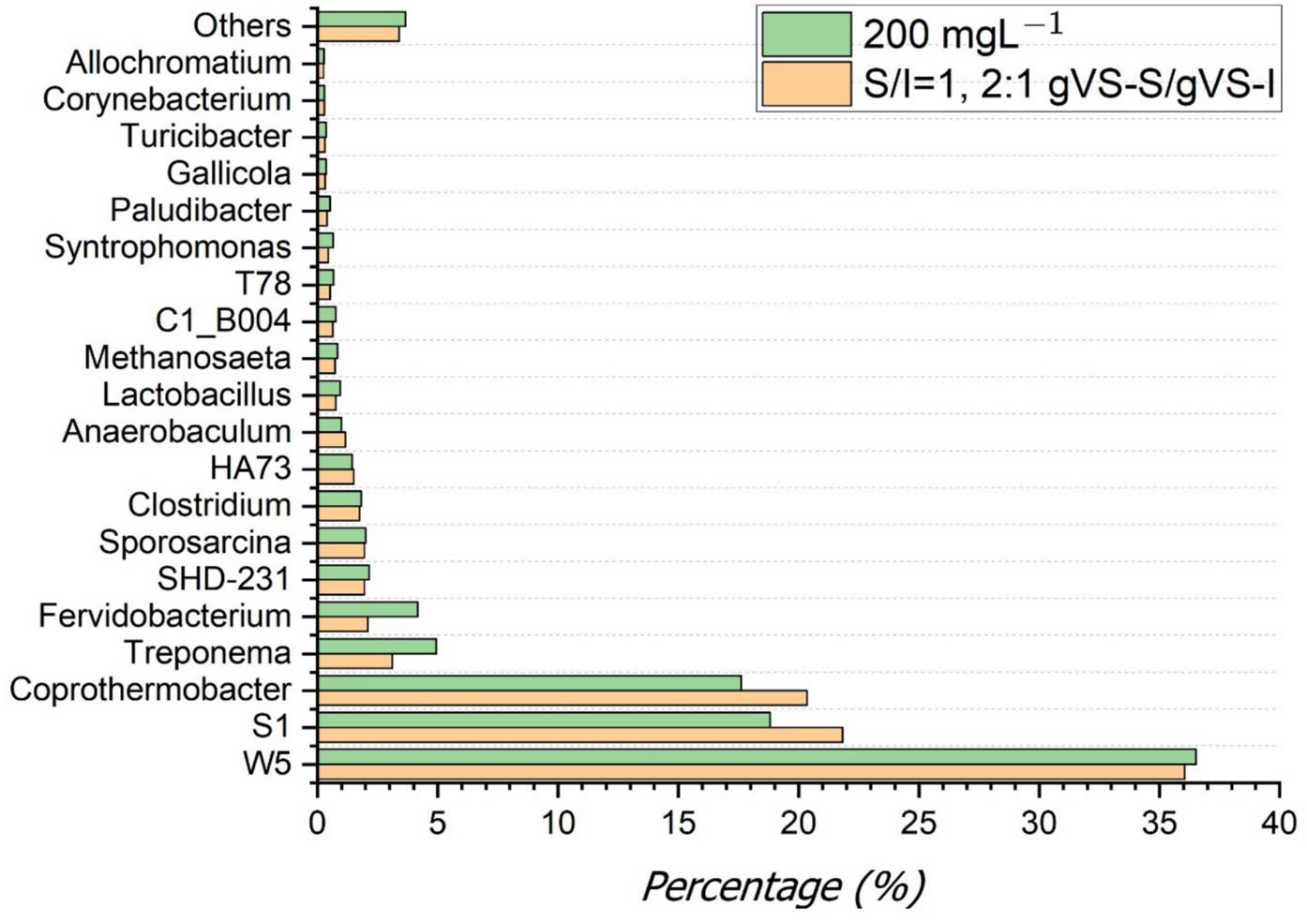
Microbial distribution in samples taken from the reactor at S/I = 1, 2:1 gVS-S/gVS-I loading ratio and microbial distribution in samples taken from the reactor at 200 mgL^−1^ magnetite concentration

To determine the microbial distribution, analysis was performed on samples obtained from reactors operated at S/I = 1 g VS-S/g VS-I with a CM:MOSW ratio of 2:1 and supplemented with 200 mg L⁻^1^ magnetite, where the highest biogas and biomethane yields were obtained (Fig. 3). In the samples taken from the S/I = 1, 2:1 gVS-S/gVS-I reactor, 36.1% *W5*, 21.8% *S1*, 20.3% *Coprothermobacter*, 3.1% *Treponema* and 2.1% *Fervidobacterium* were identified as the dominant taxa.. In the sample taken from the reactor with a loading rate of 200 mgL^−1^ magnetite, the dominant taxa were 36.5% *W5*, 18.8% *S1*, 17.6% *Coprothermobacter*, 4.9% *Treponema* and 4.2% *Fervidobacterium.* The five dominant taxa with the highest relative abundances remained largely unchanged following magnetite supplementation, indicating that magnetite did not induce major shifts in microbial community composition but rather enhanced methane production through improved functional interactions within the existing microbial consortium.

According to the microbial distribution results, *W5, S1, Coprothermobacter, Treponema and Fervidobacterium* were identified as the most prevalent taxa. Here, *W5* refers to the *Cloacimonadaceae W5* group, a fermentative bacterial cluster frequently reported in anaerobic digestion systems, whereas *S1* denotes the Bacteria *S1* group, an unclassified phylogenetic cluster also commonly enriched in anaerobic digesters.

When the microbial distribution results were examined, the first five dominant taxa (*W5, S1, Coprothermobacter, Treponema, and Fervidobacterium*) remained largely unchanged after magnetite addition. This finding indicates that magnetite did not induce a significant shift in the overall microbial community composition. Instead, the enhanced methane yield observed at 200 mgL^−1^ magnetite appears to result from functional improvements in microbial interactions rather than structural changes in community composition. In particular, magnetite can facilitate direct interspecies electron transfer (DIET) between fermentative bacteria and methanogenic archaea, thereby accelerating metabolic pathways even when the dominant taxa remain stable.

The persistence of *Cloacimonadaceae W*5 as a dominant group in both control and magnetite-amended reactors is consistent with previous studies, which reported its high relative abundance in anaerobic digestion systems despite not being classified as a core taxon (Li et al. 2022). Similarly, Shamurad et al.(Shamurad et al. 2019) found that the *Cloacimonadaceae W5* group remained dominant at different operational times, while Cazaudehore et al. (Cazaudehore et al. 2023) and Kostopoulou et al. (Kostopoulou et al. 2023) emphasized the consistent prevalence of *Coprothermobacter* across various anaerobic digestion systems. This suggests that magnetite supplementation strengthens syntrophic interactions within an already resilient microbial community rather than altering the overall community structure. In addition to taxonomic distribution, alpha diversity indices revealed moderate microbial diversity in both experimental conditions In the present study, Shannon index values of 1.92 (S/I = 1, CM:MOSW = 2:1) and 2.03 (magnetite-supplemented condition), together with Simpson indices of 0.78 and 0.79, indicate a moderately diverse and stable microbial community. Similar diversity ranges have been reported in recent anaerobic digestion studies, where Shannon indices around 2.0 and Simpson indices above 0.75 were associated with stable system operation rather than microbial restructuring (Shen et al. 2024; van Wyk et al. 2025).

Accordingly, the limited variation observed in Shannon and Simpson indices following magnetite supplementation suggests that the enhancement in methane yield was not driven by major changes in microbial population composition but rather by improved metabolic connectivity and enhanced electron transfer efficiency within the existing microbial consortium. Although methanogenic archaea play a central functional role in methane formation, they were not discussed as dominant taxa because they did not constitute a dominant fraction of the microbial community in terms of relative abundance—a pattern commonly observed in anaerobic digestion systems due to their slow growth rates and high catalytic efficiency. Consistent with this interpretation, class-level taxonomic analysis revealed the presence of archaeal groups encompassing well-characterized methanogenic genera, including *Methanosarcinia* (genus; capable of acetoclastic, hydrogenotrophic, and methylotrophic methanogenesis) as well as members of the classes *Methanobacteria* and *Methanocellia*, which are primarily associated with hydrogenotrophic methane production pathways, under both operational conditions. *Methanosarcinia* is well known for its metabolic versatility, being capable of acetoclastic, hydrogenotrophic, and methylotrophic methanogenesis, whereas *Methanobacteria* and *Methanocellia* are primarily associated with hydrogenotrophic methane production pathways. The coexistence of these archaeal classes indicates the simultaneous operation of multiple methanogenic pathways, supporting stable methane production under the applied operational conditions. Notably, the relative abundance patterns of dominant archaeal classes were largely comparable between conditions, further indicating that enhanced methane production was not driven by major shifts in archaeal community structure but by improved functional efficiency within the existing methanogenic consortium.

## Conclusion

This study demonstrates that the effectiveness of anaerobic digestion enhancement strategies is governed not merely by the presence of conductive additives, but by their ability to modulate interspecies electron transfer under specific substrate loading conditions. The results indicate that magnetite does not universally stimulate methane production; rather, its beneficial impact emerges within a narrow operational window defined by the substrate-to-inoculum ratio and substrate composition.

The identification of an optimal interaction window (S/I = 1 gVS-S/gVS-I, CM:MOSW = 2:1, and 200 mg L⁻^1^ magnetite) suggests that magnetite primarily facilitates direct interspecies electron transfer (DIET) between syntrophic bacteria and methanogenic archaea. Under these conditions, conductive magnetite particles likely act as electron conduits, reducing the reliance on diffusible electron carriers such as hydrogen and formate. This mechanism enhances thermodynamic favorability and accelerates syntrophic metabolism, thereby improving methane yield and solids degradation.

Importantly, microbial community analysis revealed no significant shifts in dominant taxa following magnetite supplementation. This stability indicates that performance enhancement is not driven by microbial selection or enrichment, but by functional intensification of the existing microbial consortia. Magnetite appears to strengthen metabolic coupling among established populations by lowering electron transfer resistance and increasing electron flux efficiency within the anaerobic network.

At suboptimal substrate loading conditions, however, the conductive properties of magnetite did not translate into performance gains. Excessive or insufficient substrate-to-inoculum ratios likely disrupted the balance between hydrolytic, acidogenic, and methanogenic processes, thereby constraining the potential benefits of enhanced electron transfer. These observations highlight that DIET-based enhancement is inherently conditional and strongly dependent on maintaining favorable process kinetics and redox balance.

Collectively, these findings provide mechanistic insight into how magnetite enhances anaerobic digestion of nitrogen-rich wastes. They emphasize that dosage control and substrate loading are decisive parameters governing whether conductive additives promote efficient electron transfer or remain functionally ineffective. From a process design perspective, aligning conductive material supplementation with optimized operational conditions enables improved reactor stability and methane productivity without altering microbial community structure.

### Statement of novelty

This study offers a novel mechanistic framework by jointly evaluating substrate-to-inoculum ratio and magnetite supplementation in the co-digestion of chicken manure and municipal organic solid waste. Unlike prior studies that assess conductive additives or substrate loading in isolation, this work demonstrates that magnetite-driven performance enhancement is contingent upon substrate-induced metabolic balance. By integrating process performance metrics with microbial community stability, the study elucidates that conductive additives enhance anaerobic digestion primarily through DIET-mediated functional optimization rather than microbial restructuring. This mechanistic perspective advances current understanding of anaerobic digestion optimization, particularly for nitrogen-rich substrates.

### Statement of industrial relevance

The mechanistic insights derived from this study have direct implications for full-scale biogas plants treating nitrogen-rich waste streams. The identification of an optimal substrate-to-inoculum ratio combined with a targeted magnetite dosage provides a practical strategy to enhance methane productivity by reinforcing interspecies electron transfer pathways. Importantly, the observed improvements arise without necessitating changes in reactor configuration or microbial inocula, making this approach both scalable and economically viable. By promoting functional efficiency through DIET rather than microbial replacement, the findings support robust and resilient waste-to-energy systems aligned with circular economy and sustainable energy objectives.

## Data Availability

No datasets were generated or analysed during the current study.
